# Lapping Quality Prediction of Ceramic Fiber Brush Based on Gaussian-Restricted Boltzmann Machine

**DOI:** 10.3390/ma15217805

**Published:** 2022-11-04

**Authors:** Xiuhua Yuan, Chong Wang, Mingqing Li, Qun Sun

**Affiliations:** School of Mechanical and Automotive Engineering, Liaocheng University, Liaocheng 252000, China

**Keywords:** ceramic fiber brush, aluminium alloy, lapping, multi-layer neural network, gaussian restricted boltzmann machine

## Abstract

Although ceramic fiber brushes have been widely used for deburring and surface finishing, the associated relationship between process parameters and lapping quality is still unclear. In order to optimize the lapping process of ceramic fiber brushes, this paper proposes a multi-layer neural network based on the Gaussian-restricted Boltzmann machine (GRBM), and verified its prediction effectiveness. Compared with a traditional back-propagation neural network, its prediction error was reduced from 7.6% to 4.5%, and the determination coefficient was increased from 0.96 to 0.98, respectively. The comparison results showed that the proposed model can better grasp the relationship between process parameters and machining quality, which can be used as a decision-making foundation for lapping-process optimization.

## 1. Introduction

**Figure 1:**
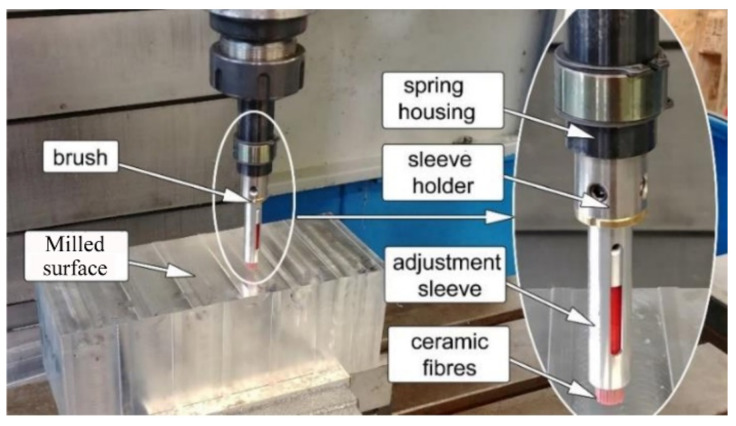
Lapping experiment in CNC machine tool.

Although abrasive filament brushes have been successfully used for manual and automated applications, including burr removal [1,2], edge rounding [3], polishing [4,5], and frosted glass [6,7], they show poor consistency of machining quality due to the fast wear of the nylon filament [8]. To improve the disadvantage of existing brush-type surface finishing tools, the ceramic fiber brush has been developed and applied to deburring parts, showing better durability and machinability [9,10]. As shown in Figure 1 [11], ceramic fiber brushes have recently been studied to explore the possibility of mirror finishing based on their high machinability [12,13]. However, the associated relationship between process parameters and lapping quality is still not clear. 

In the field of machining processes, back-propagation (BP) neural networks [14,15,16,17,18,19,20] have been widely used to predict the value of machining quality. During the above training process, problems of over fitting and gradient disappearance [21,22] are encountered with the increasing of hidden layers. In order to overcome the above problem, the restricted Boltzmann machine [23,24] was applied to pre-training the multi-layer neural networks, and provided the initial values of weight. After pre-training the multi-layer neural networks by binary restricted Boltzmann machine, the grade of milling quality was accurately classified [25]. However, in order to optimize the milling process, it is urgent to predict an accurate value of milling quality. Moreover, the continuous values of machining parameters cannot be directly input into the binary-restricted Boltzmann machine.

The Gaussian-restricted Boltzmann machine (GRBM) is a special form of logarithmic linear Markov random field, and assumes that the conditional probability of the visible layer element follows Gaussian distribution, which can model the continuous value of process parameters [26]. On the other hand, 7xxx-series aluminum alloy is an important aviation aluminum material due to its high strength, and is widely applied in wing surfaces, body skin, forgings, reinforcements, etc. After milling, a ceramic fiber brush can be applied to remove burrs and tool marks [27,28,29] due to the milling vibration, especially in thin-wall parts. In order to reveal the associated relationship between process parameters and lapping quality and accurately predict the lapping quality, this paper carried out a brush-lapping experiment on aluminum alloy, constructed a multi-layer neural network based on GRBM, and verified its prediction performance.

## 2. Prediction Model of Brush-Lapping Quality

### 2.1. Lapping Experiment of Ceramic Fiber Brush

**Figure 2:**
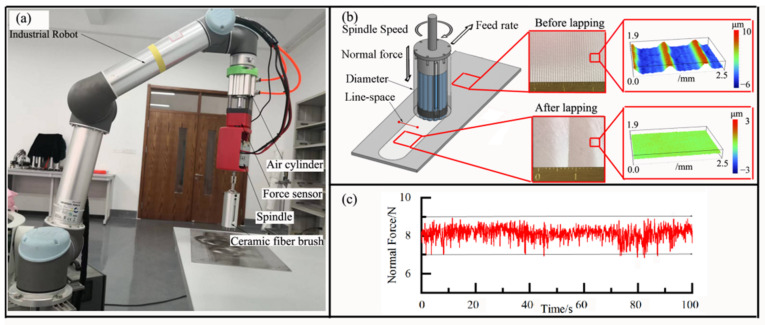
Robotic automatic lapping platform (**a**), process parameters (**b**), and constant of normal force (**c**).

As shown in Figure 2a, the lapping experiment was carried out on a self-made robotic automatic platform, which was composed of an industrial robot, robot controller, pneumatic cylinder, force sensor, motor spindle, ceramic fiber brush, etc. A six-axis robot with force control was employed as the industrial robot, and a ceramic brush with white fibers (Xebec, Tokyo, Japan) was used for lapping experiment. A single fiber was composed of about 1000 ultrafine fibers with a diameter of several microns, composed of aluminum oxide. The ultrafine fiber works as self-sharpening abrasive, and can endure temperatures up to 150 °C. During the lapping experiment, the normal force of the ceramic fiber brush was tested and fed back to the platform using the force sensor (STC-10kg, Celtron, Allendale, MI, USA). In addition, the normal force of the ceramic fiber brush can be maintained, and adjusted through the pressure of the pneumatic cylinder, whose variation was lower than 2.1 N (Figure 2c).

The material of the specimen was 7075 aluminum alloy, which is widely used in the aerospace industry. As shown in Figure 2b, all specimens were milled with ball-end cutter (diameter: 20 mm) before the lapping experiment, and their surface roughness was 4.5 μm. In the lapping process, the process parameters are normal force, spindle speed, feed speed, polishing line spacing, and ceramic fiber brush diameter. In this paper, the line-space was expressed as the pitches between first and second lapping path, and its value was the ratio of pitches to the brush diameter. In order to train the multi-layer neural network, lapping experiments were carried out under different process parameters. As shown in Table 1, the brush diameter was divided into three levels, and the other process parameters were divided into four levels. Thus, the total number of training samples was 768, and some representative samples of the dataset are shown in Table A1. After the lapping experiment, a stylus-type profilometer (TIME 3200, Beijing, China) was used to measure the lapped surface, and the arithmetic mean roughness (Ra) was selected to evaluate the surface quality of the lapped surface. The stylus-type profilometer had a contact probe sensor, and its sample length was set to 2.5 mm. During the measuring, the movement direction of the probe was perpendicular to the lapping feed direction (Figure A1). The Ra was measured at five evenly spaced locations (P1, P2, P3, P4, P5) along the length of the lapping feed direction, and the mean value of the measured values at five locations was the final Ra at a certain process parameter. The surface topography of workpiece was measured by a Wyko NT9800 optical profiler (Veeco Instruments Inc., New York, NY, USA).

### 2.2. Prediction Model Based on Gaussian-Restricted Boltzmann Machine

#### 2.2.1. Structure Design of the Prediction Model

**Figure 3:**
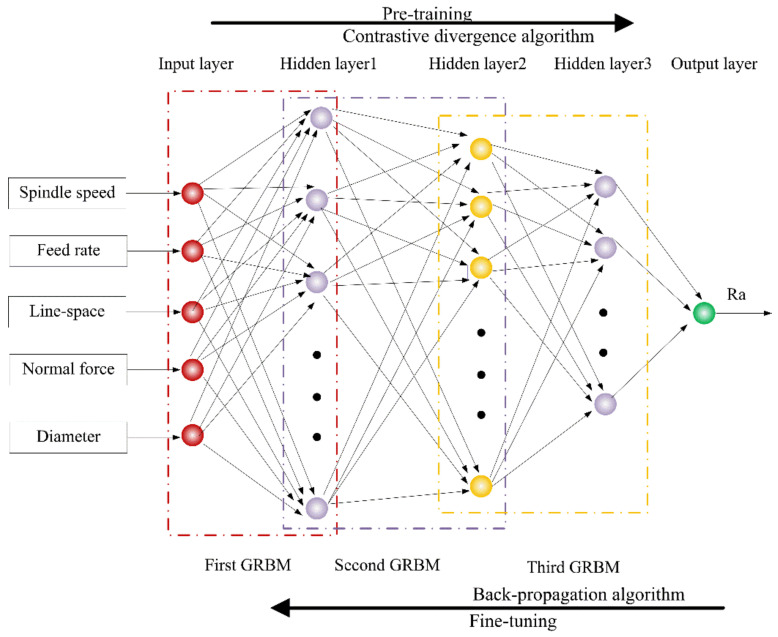
The structure of the prediction model.

**Figure 4:**
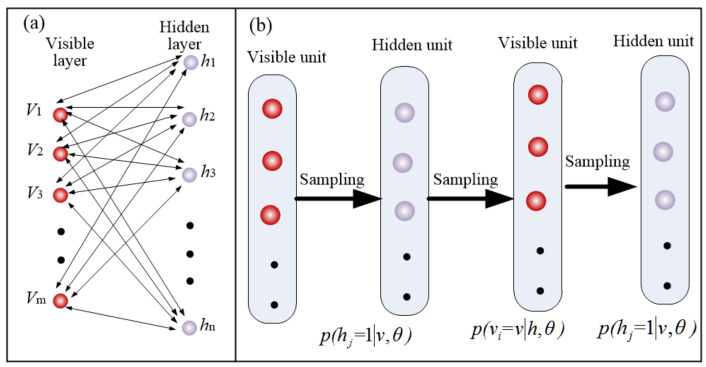
Diagram of Gaussian-restricted Boltzmann machine (**a**), and pre-training process (**b**).

As shown in Figure 3, the topological structure of the prediction model was mainly composed of the input layer, three hidden layers, and the output layer. The five neurons in the input layer corresponded to the process parameters: spindle speed, feed rate, line-space, normal force, brush diameter. The number of neurons in the three hidden layers corresponded to 16, 12, 8, respectively. The neuron of the output layer corresponded to the arithmetic mean deviation of contour (Ra). The hidden layers were composed of three GRBMs, which served for detecting the data features from the visible layer based on the connection weights. In order to keep all the data consistent, the experiment data were normalized to the interval of [0, 1]. As shown in Figure 4a, the GRBM was a typical two-layer neural network, which contained a visible layer and a hidden layer. The visible layer of the following GRBM received the outputs of the hidden layer of the previous GRBM.

The training process of the proposed model consisted of two stages: unsupervised pre-training and supervised fine-tuning, which correspond to the GRBM and BP algorithm, respectively. In the first stage, all GRBMs were trained layer by layer according to above using a contrastive divergence algorithm (Figure 4b), and the initial weights, which represent the lapping features, were obtained. In the second stage, according to the label of the output data, the prediction model was treated as a traditional multi-layer neural network, and the gradient-descent algorithm was used to fine tune the weights.

#### 2.2.2. Pretraining Process of Gaussian-Restricted Boltzmann Machine

Since the process parameters are continuously distributed, the neurons in the visible layer of the restricted Boltzmann machine must have continuous values. Therefore, the GRBM was applied to pre-training the prediction model. As shown in Figure 4, there were undirected connections between all units of the visible layer and hidden layer, and no connection between units at the same layers. For the GRBM, its energy function was shown as follows:

(1) $$E(v,h\left|\theta \right.)=\sum_{i=1}^{m}\frac{{\left(v_{i}-a_{i}\right)}^{2}}{2\sigma_{i}^{2}}-\sum_{j=1}^{n}h_{j}b_{j}-\sum_{i=1}^{m}\sum_{j=1}^{n}\frac{w_{ij}v_{i}h_{j}}{\sigma_{i}^{2}}$$

where $\theta =\left[w_{ij},\;a_{i},\;b_{j},\;\sigma_{i}\right]$ was the parameters set to be learned, $v_{i}$ and $h_{j}$ were the neurons of the visible layer and hidden layer, respectively, $w_{ij}$ was connection weight between the hidden layer and visible layer, $a_{i}$ and $b_{j}$ were the bias of the visible layer and hidden layer, respectively, m and n were the number of visible units and hidden units, respectively, and $\sigma_{i}^{2}$ was the variance of Gaussian distribution of visible units. The activation probability of the hidden unit *j* can be deduced as follows:

(2) $$p(h_{j}=1\left|v,\theta \right.)=\frac{1}{1+e^{-(b_{j}+\sum_{i=1}^{m}\frac{w_{ij}v_{i}}{\sigma_{i}^{2}})}}\;=sigmoid(b_{j}+\sum_{i=1}^{m}\frac{w_{ij}v_{i}}{\sigma_{i}^{2}})$$

The activation probability of visible units *i* can be deduced as follows:

(3) $$p(v_{i}=v\left|h,\theta \right.)=\exp(\frac{a_{i}+\sum_{j=1}^{n}w_{ij}h_{j}}{\sigma_{i}^{2}})\;=N(a_{i}+\sum_{j=1}^{n}w_{ij}h_{j},\sigma_{i}^{2})$$

where $N(\mu ,\sigma^{2})$ was Gaussian distribution with mean μ and variance $\sigma^{2}$. The partial derivatives of log-likelihood can be expressed as follows:

(4) $$\frac{\partial L(\theta \left|v\right.)}{\partial w_{ij}}={\langle \frac{v_{i}}{\sigma_{i}^{2}}h_{j}\rangle }_{data}-\;{\langle \frac{v_{i}}{\sigma_{i}^{2}}h_{j}\rangle }_{model}$$

(5) $$\frac{\partial L(\theta \left|v\right.)}{\partial a_{i}}={\langle \frac{v_{i}}{\sigma_{i}^{2}}\rangle }_{data}-\;{\langle \frac{v_{i}}{\sigma_{i}^{2}}\rangle }_{model}$$

(6) $$\frac{\partial L(\theta \left|v\right.)}{\partial b_{j}}={\langle h_{j}\rangle }_{data}-\;{\langle h_{j}\rangle }_{model}$$

(7) $$\frac{\partial L(\theta \left|v\right.)}{\partial \sigma_{i}}={\langle \frac{{\left(v_{i}-a_{i}\right)}^{2}}{\sigma_{i}^{3}}-\sum_{j=1}^{n}h_{j}\frac{\left(w_{ij}v_{i}\right)}{\sigma_{i}^{3}}\rangle }_{data}-\;{\langle \frac{{\left(v_{i}-a_{i}\right)}^{2}}{\sigma_{i}^{3}}-\sum_{j=1}^{n}h_{j}\frac{\left(w_{ij}v_{i}\right)}{\sigma_{i}^{3}}\rangle }_{model}$$

where ${\langle •\rangle }_{data}$ and ${\langle •\rangle }_{model}$ were referred to the inner product of original data and reconstructed data, respectively. According to the contrastive divergence algorithm, the parameters update rules were written as follows:

(8) $$w_{ij}(\tau +1)=w_{ij}(\tau )+\eta ({\langle \frac{v_{i}}{\sigma_{i}^{2}}h_{j}\rangle }_{data}-{\langle \frac{v_{i}}{\sigma_{i}^{2}}h_{j}\rangle }_{model})$$

(9) $$a_{i}(\tau +1)=a_{i}(\tau )+\eta ({\langle \frac{v_{i}}{\sigma_{i}^{2}}\rangle }_{data}-{\langle \frac{v_{i}}{\sigma_{i}^{2}}\rangle }_{model})$$

(10) $$b_{j}(\tau +1)=b_{j}(\tau )+\eta ({\langle h_{j}\rangle }_{data}-{\langle h_{j}\rangle }_{model})$$

(11) $$\sigma_{i}(\tau +1)=\sigma_{i}(\tau )+\eta ({\langle \frac{{\left(v_{i}-a_{i}\right)}^{2}}{\sigma_{i}(\tau {)}^{3}}-\sum_{j=1}^{n}h_{j}\frac{\left(w_{ij}v_{i}\right)}{\sigma_{i}(\tau {)}^{3}}\rangle }_{data}-\;{\langle \frac{{\left(v_{i}-a_{i}\right)}^{2}}{\sigma_{i}(\tau {)}^{3}}-\sum_{j=1}^{n}h_{j}\frac{\left(w_{ij}v_{i}\right)}{{(}_{i})\tau {(}^{3}}\rangle }_{model})$$

where η was a learning step. The Python scripting language and TensorFlow open source platform were used to construct the proposed model, and the main pre-training steps are shown in Figure A2.

#### 2.2.3. Fine-Tuning Process 

After pre-training by GRBM, the multi-layer neural network was fine-tuned by back-propagation algorithm. That is, the initial value of weight was provided by previous step of GRBM. During the fine-tuning process, the activation functions of hidden-layer and output-layer neurons were a sigmoid function and a pure line function, respectively. The sigmoid function was as follows:

(12) $$f(x)=\frac{1}{1+e^{-x}}$$

The mean square error function (*MSE*) was selected as the loss function, shown as follows:

(13) $$MSE=\frac{1}{n}\sum_{i=1}^{n}{\left({\hat{y}}_{i}-y_{i}\right)}^{2}$$

where *n* was the number of the batch size, and ${\hat{y}}_{i}$ and $y_{i}$ were the predicted and actual values for sample *i*. The weight was then updated by the steepest descendent method, and moved toward a better solution, which was as follows:

(14) $$w^{\left(k+1\right)}=w^{\left(k\right)}+\eta \frac{\partial (MSE)}{\partial w}$$

where the *k* and η were the interaction period and step parameter of the correction, respectively. If the mean square error of network training was less than the initial minimum error target or the number of learning periods exceeded the initial maximum set value, the iteration cycle would stop.

## 3. Results and Discussion

### 3.1. The Experiment Result

**Figure 5:**
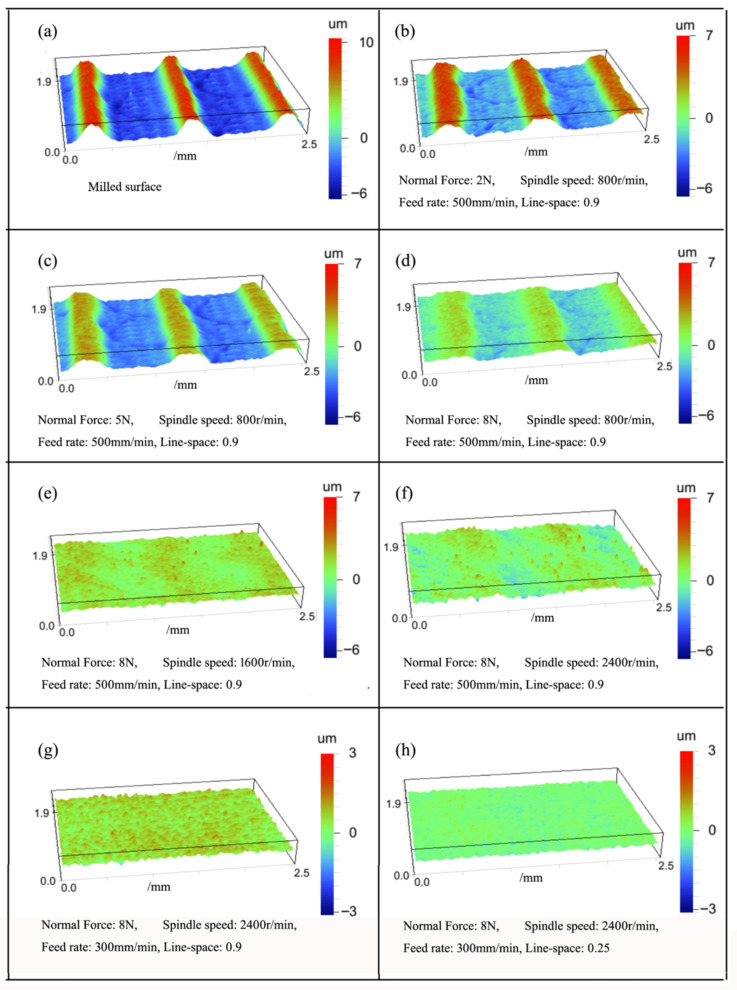
Variations of surface topography under different process parameters.

The relationship between process parameters and surface quality can be revealed through the analysis of the experimental data. Figure 5 depicts the surface morphology of aluminum alloy before and after the lapping experiment using ceramic fiber brush (diameter: 40 mm). As shown in Figure 5a, the surface morphology before lapping showed the parallel peaks and valleys, which was obviously caused by the translation of the workpiece and the rotational motion of the ball-end cutter. As shown in Figure 5b, the surface roughness after lapping changed from Ra = 4.5 μm to Ra = 2.3 μm, and the peak was reduced due to the cutting of the abrasive grain. As shown in Figure 5c,d, the majority of surface peaks were removed when the normal force was further increased to 8 N. As shown in Figure 5e,f, almost all the surface peaks were removed, and the surface roughness was reduced to Ra = 0.68 μm, when the spindle speed was further increased to 2400 r/min. As shown in Figure 5g,h, the surface became smoother, and the minimum value of surface roughness was reduced to Ra = 0.24 μm, when the feed rate and line-space were further decreased. The above results indicate that these process parameters have different effects on the lapping quality.

ANOVA (Analysis of variance) is a statistical technique used to test the significance of factors by comparing a mean square to an estimate of the experimental flaw at a certain level of confidence. As shown in Table 2, a Taguchi L_16_ orthogonal experimental table involving four factors at four levels and one factor at three levels was constructed. All standard deviations of Ra were below 0.049, which indicated that the measured value of Ra can better reflect surface roughness of lapped surface at certain process parameters. As shown in Table 3, the *p*-values of ANOVA were 0.003, 0.004, 0.011, 0.031, and 0.013, respectively. All *p*-values were below 0.05, which indicated that the five process parameters had a significant effect on the lapped surface.

**Figure 6:**
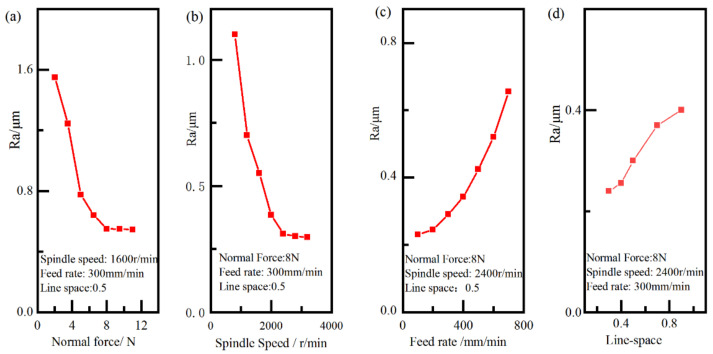
Main effect plots of process parameters on the surface roughness.

The effect of process parameters on the surface roughness using single-factor analysis was shown in Figure 6. As shown in Figure 6a, the surface roughness first declined rapidly, and then decreased slowly with the increase in the normal force. This can be explained in that the larger normal force causes abrasive grain to press against the workpiece’s surface, resulting in better surface lapping. From Figure 6b, the surface roughness first rapidly declined, and then slowly decreased with the increase in the spindle speed. This was because the higher spindle speed can effectively increase the kinetic energy of abrasive grain and reduce uplift height by the abrasive plough, resulting better surface quality. From Figure 6c,d, the surface roughness slowly increased first, and then rapidly increased with the increase in the feed rate and line-space. This can be explained in that the time of lapping can be increased with a decrease in feed rate and line-space, resulting better surface quality.

### 3.2. Network Training and Prediction Performance

**Figure 7:**
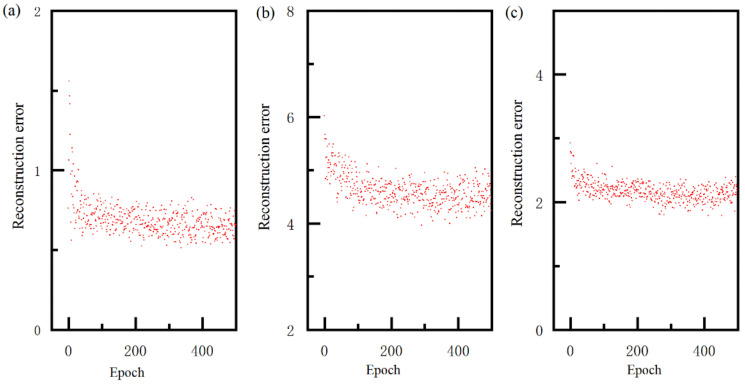
Reconstruction error of first GRBM (**a**), second GRBM (**b**) and third GRBM (**c**).

The main purpose of this section was to evaluate the multi-layer neural network pretrained by GRBM and predict the lapping quality. The structure of the multi-layer neural network was 5-16-12-8-1, and the training dataset was 768 samples. Without losing objectivity, the number of iterations and the learning rate in unsupervised pre-training stage were 500 and 0.01, respectively. In the field of statistical thermodynamics, the Boltzmann machine was used to study the probability distribution of gas molecules in thermal equilibrium. In order to analyze the energy change in GRBM, this paper calculated the reconstruction error ($\langle \frac{v_{i}}{\sigma_{i}^{2}}h_{j}\rangle {}_{data}-\langle \frac{v_{i}}{\sigma_{i}^{2}}h_{j}\rangle {}_{model}$) during the unsupervised pre-training stage, which was shown in Figure 7. As shown in Figure 7a, the reconstruction error at initial phase was large, which can be explained in that the bias ($a_{i}$, $b_{j}$) and connection weight ($w_{ij}$) were randomly generated. The reconstruction error decreased with the increase in epoch, which implied that the energy of the total particles was decreased. Finally, the reconstruction error was minorly changed when the epoch was larger than 100, which implies that the total particles were in a stable state. These three Boltzmann machines showed a similar trend of reconstruction error with the increase in epoch, which was similar to that reported in another work [30]. From the above analysis, it can be seen that the GRBM shows better reconstruction ability, and can model complex hierarchical data. After the unsupervised pre-training stage, the weights of the multi-layer neural network were initialized.

**Figure 8:**
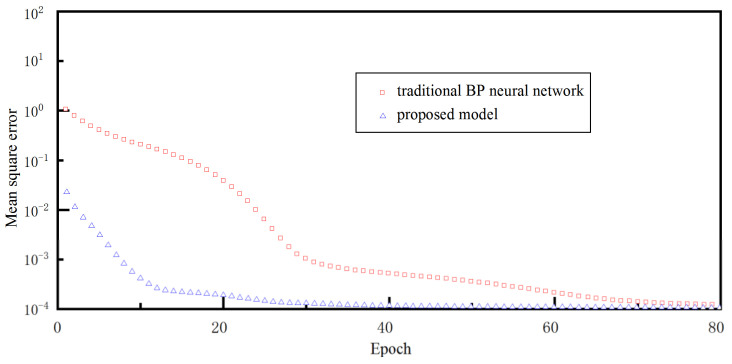
The output error in the iterative optimization process.

During the fine-tuning stage, the transfer functions of the hidden-layer and output-layer neurons were a sigmoid function and a pure line function, respectively. In order to evaluate its training performance, the multi-layer neural network was also only trained by the standard BP algorithm, that is, the weight of multi-layer neural network was randomly initialized, rather than the value of GRBM. The mean square error ($\frac{1}{n}\sum_{i=1}^{n}{\left({\hat{y}}_{i}-y_{i}\right)}^{2}$, where ${\hat{y}}_{i}$ and $y_{i}$ were the predicted and actual values for sample *i*) was applied to evaluate the prediction accuracy during the iterative optimization process. Figure 8 shows the change in the iteration error with epoch during the fine-tuning stage. The iteration error of proposed model decreased sharply at the early stage, and then decreased slowly when the iterations exceeded 10. The iteration error decreased to 0.0001 and almost ceased to change when the epochs exceeded 40. Compared with the BP neural network, the proposed model has little iteration error at the initial stage, and converges quickly to a minimal value of output error. From the above analysis, it can be seen that the GRBM can provide appropriate weights to the multi-layer neural network, and avoid excessive training in the fine-tuning stage.

**Figure 9:**
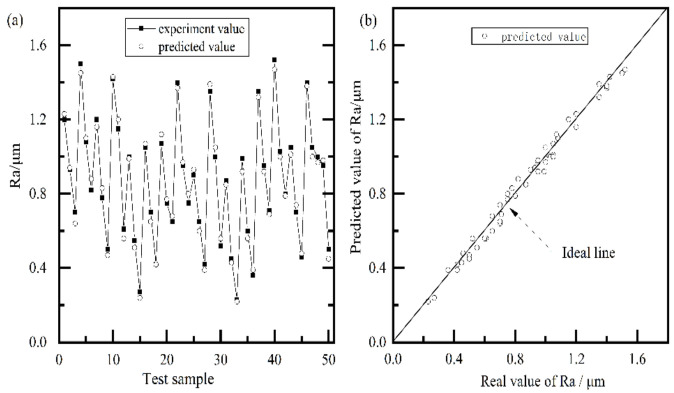
Testing result (**a**) and regression plot of predicted value and experiment value (**b**).

**Figure A1:**
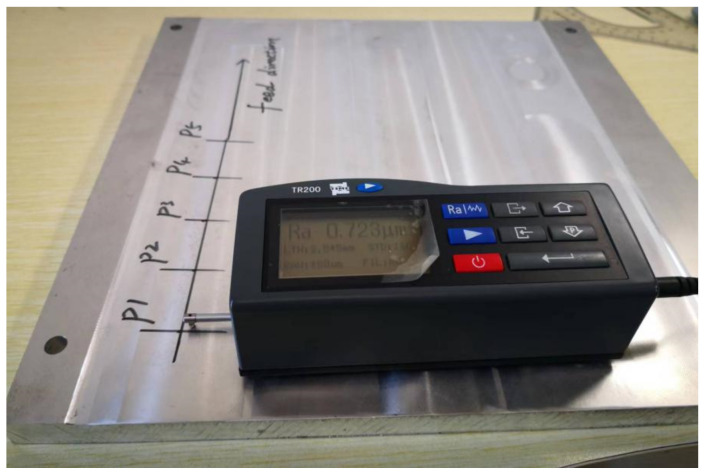
The measurement experiment for lapping quality.

**Figure A2:**
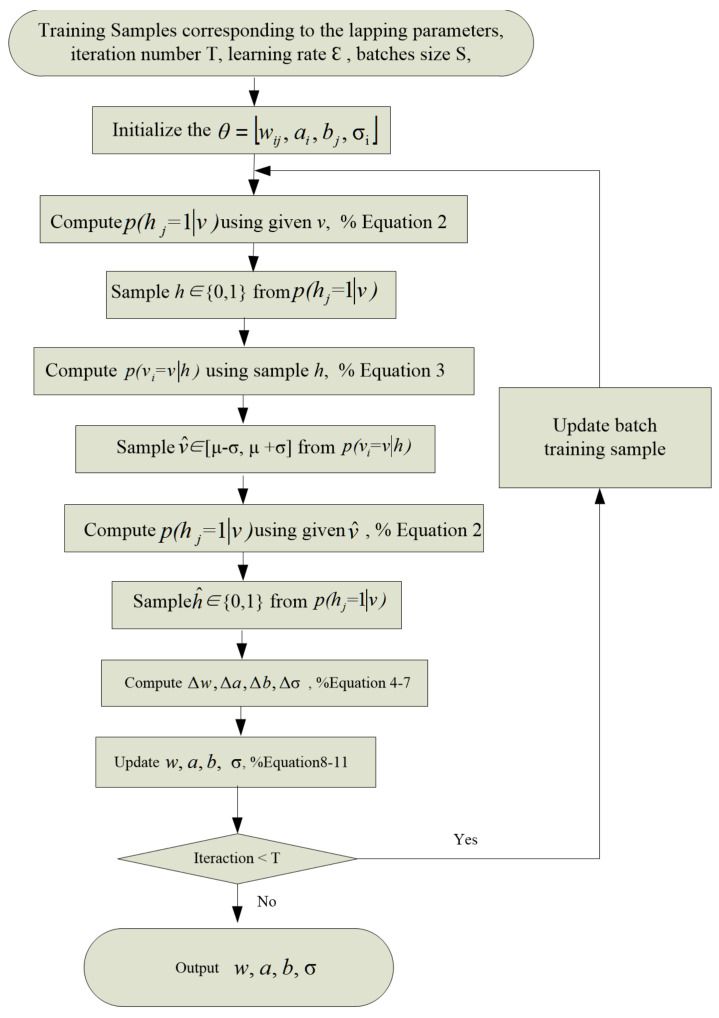
The pre-training process by GRBM.

After training process, the testing samples (Table A2) were used to test the prediction performance, and the mean absolute percentage error (*MAPE* = $\frac{1}{n}\sum_{i=1}^{n}\left|\frac{{\hat{y}}_{i}-y_{i}}{y_{i}}\right|\times 100\%$, where ${\hat{y}}_{i}$ and $y_{i}$ were the predicted value, actual value, respectively) and the determination coefficient (*R*^2^ =$1-\frac{\sum_{i=1}^{n}{\left({\hat{y}}_{i}-y_{i}\right)}^{2}}{\sum_{i=1}^{n}{\left(y_{i}-\bar{y}\right)}^{2}}$, where ${\hat{y}}_{i}$, $y_{i}$ and $\bar{y}$ were the predicted value, experiment value, and mean average value, respectively) were applied to evaluate the prediction accuracy. It should be noted that the testing samples were independent of training data, which is universally emphasized in applications of neural networks. Figure 9a showed the comparison between the experiment value and predicted value, and the predicted value was also shown in Table A2. It can be observed that the predicted value can almost fit with the experiment value curve, and follows the variation trend of the experiment data well. Figure 9b shows the regression plot of predicted value and experiment value. The closer the points were to the perfect line, the better the prediction ability of the proposed model. It was clear that the predicted values were in good agreement with the experiment values, as all the points were located approximately on the ideal line. The *MAPE* and *R*^2^ of the proposed model were 4.5% and 0.98, respectively. The traditional multi-layer neural network was also constructed, that is, the weight was randomly generated, and then only trained by the standard BP algorithm. Its *MAPE* and *R*^2^ were 7.6% and 0.96, respectively, which was similar to those of a previous work [20,21,22,31]. From the above data analysis, the proposed model has better generalization performance than the traditional neural network. This may be due to the face that the GRBM provided the appropriate weights [30], resulting in better prediction performance. Thus, a multi-layer neural network based on GRBM can be used as a decision-making base for lapping-process optimization.

## 4. Conclusions

Although ceramic fiber brushes have been widely used in deburring and surface finishing, the associated lapping process is still not clear. The novelty of this study is in introducing a multi-layer neural network pretrained by GRBM for lapping quality prediction. A lapping experiment was carried out, and the following conclusions were drawn:

1. During the brush lapping of aluminum alloy, the surface roughness decreased with the increasing of normal force and spindle speed, and increased with the increasing of the feed rate and line-space. Moreover, the minimum of surface roughness can be reduced to 0.24 μm.
2. In the unsupervised pre-training stage, the reconstruction error of GRBM decreased with an increase in epoch, and minorly changed when the epoch was larger than the 100th.
3. Compared with the BP neural network, the prediction error of the proposed model was reduced from 7.6% to 4.5%, and the *R*^2^ was increased from 0.96 to 0.98, respectively, showing the better performance of generalization.

This paper aims to develop an intelligent robot system for lapping workpieces with ceramic fiber brushes. In order to better apply the proposed model to an actual lapping environment, the next step is to optimize the architecture of the multi-layer neural network, and construct a lapping database about metals (such as magnesium alloy, aluminum alloy, cast iron, steel, and titanium alloy).

## Figures and Tables

**Table 1:** The Process Parameters and Their Levels.

Level	Brush Diameter /mm	Spindle Speed /r/min	Feed Rate /mm/min	Line-Space /Pitches/Diameter	Normal Force /N
1	25	800	100	0.25	2
2	40	1600	300	0.50	5
3	60	2400	500	0.75	8
4	-	3200	700	0.9	11

**Table 2:** L_16_ Orthogonal Experiment.

No	Process Parameters					Replicate					Average Ra µm	Standard Deviation
	Normal Force N	Spindle Speed r/min	Feed Rate mm/min	Line-Space	Diameter mm	Ra 1 µm	Ra 2 µm	Ra 3 µm	Ra 4 µm	Ra 5 µm		
1	2	800	100	0.25	25	1.94	1.95	2.07	2.05	1.99	2.00	0.039
2	2	1600	500	0.90	40	1.81	1.75	1.80	1.84	1.77	1.79	0.044
3	2	2400	700	0.50	60	1.32	1.36	1.33	1.39	1.35	1.35	0.041
4	2	3200	300	0.75	25	1.15	1.12	1.08	1.16	1.09	1.10	0.045
5	5	800	300	0.50	40	1.43	1.48	1.45	1.49	1.45	1.46	0.034
6	5	1600	700	0.75	25	0.86	0.87	0.93	0.95	0.89	0.90	0.049
7	5	2400	500	0.25	25	0.81	0.76	0.80	0.85	0.82	0.81	0.034
8	5	3200	100	0.90	60	0.35	0.37	0.41	0.44	0.38	0.39	0.028
9	8	800	500	0.75	60	1.27	1.23	1.21	1.31	1.23	1.25	0.009
10	8	1600	100	0.50	25	0.50	0.51	0.59	0.58	0.57	0.45	0.038
11	8	2400	300	0.90	25	0.60	0.60	0.60	0.64	0.60	0.61	0.025
12	8	3200	700	0.25	40	0.40	0.46	0.53	0.52	0.47	0.48	0.031
13	11	800	700	0.90	25	0.91	0.88	0.90	0.95	0.90	0.91	0.036
14	11	1600	300	0.25	60	0.56	0.53	0.55	0.59	0.57	0.56	0.034
15	11	2400	100	0.75	40	0.26	0.27	0.32	0.34	0.31	0.30	0.026
16	11	3200	500	0.50	25	0.42	0.47	0.54	0.56	0.51	0.50	0.034

**Table 3:** The ANOVA Result for the Ra.

Source	DOF	Sum of Squares	Variance	F-Value	*p*	Significant
**Normal force**	3	2.367	0.789	63108	0.003	Yes
**Spindle speed**	3	1.438	0.479	38345	0.004	Yes
**Feed rate**	3	0.161	0.054	4296	0.011	Yes
**Line-space**	3	0.021	0.007	566	0.031	Yes
**Diameter**	2	0.077	0.038	3073	0.013	Yes
**Error**	1	0.00012	0.00012	-	-	-
**Total**	16	18.126	-	-	-	-

## Data Availability

Not applicable.

## Appendix A

**Table A1:** The representative experimental results for training.

No	Process parameters					Measure Value Ra /μm
	Diameter /mm	Spindle Speed /r/min	Feed Rate /mm/min	Line-Space	Normal Force /N	
1	25	1600	300	0.5	2	1.32
2	25	1600	300	0.5	5	0.55
3	25	1600	300	0.5	8	0.42
4	25	1600	300	0.5	11	0.60
5	25	800	300	0.5	8	1.25
6	25	2400	300	0.5	8	0.29
7	25	3200	300	0.5	8	0.32
8	25	2400	700	0.5	8	0.86
9	25	2400	500	0.5	8	0.59
10	25	2400	100	0.5	8	0.29
11	25	2400	300	0.25	8	0.32
12	25	2400	300	0.7	8	0.40
13	25	2400	300	0.9	8	0.52
14	40	1600	300	0.5	2	1.55
15	40	1600	300	0.5	5	0.78
16	40	1600	300	0.5	8	0.55
17	40	1600	300	0.5	11	0.54
18	40	800	300	0.5	8	1.1
19	40	2400	300	0.5	8	0.31
20	40	3200	300	0.5	8	0.29
21	40	2400	700	0.5	8	0.66
22	40	2400	500	0.5	8	0.43
23	40	2400	100	0.5	8	0.28
24	40	2400	300	0.25	8	0.24
25	40	2400	300	0.7	8	0.37
26	40	2400	300	0.9	8	0.40
27	60	1600	300	0.5	2	1.62
28	60	1600	300	0.5	5	1.23
29	60	1600	300	0.5	8	0.80
30	60	1600	300	0.5	11	0.65
31	60	800	300	0.5	8	1.0
32	60	2400	300	0.5	8	0.38
33	60	3200	300	0.5	8	0.31
34	60	2400	700	0.5	8	0.93
35	60	2400	500	0.5	8	0.78
36	60	2400	100	0.5	8	0.29
37	60	2400	300	0.25	8	0.32
38	60	2400	300	0.7	8	0.45
39	60	2400	300	0.9	8	0.55

**Table A2:** The experiment results for testing.

No	Process Parameters					Measured Value Ra/μm	Predicted Value Ra/μm
	Diameter/mm	Spindle Speed /r/min	Feed Rate /mm/min	Line-Space	Normal Force /N		
1	25	1200	200	0.3	3	1.20	1.23
2	25	1200	200	0.3	6	0.93	0.94
3	25	1200	200	0.3	9	0.70	0.64
4	25	1200	400	0.3	3	1.50	1.45
5	25	1200	400	0.3	6	1.08	1.10
6	25	1200	400	0.3	9	0.82	0.88
7	25	2000	200	0.6	3	1.20	1.16
8	25	2000	200	0.6	6	0.78	0.83
9	25	2000	200	0.6	9	0.50	0.47
10	25	2000	400	0.6	4	1.42	1.43
11	25	2000	400	0.6	6	1.15	1.20
12	25	2000	400	0.6	9	0.61	0.56
13	25	2800	200	0.3	4	1.00	0.99
14	25	2800	200	0.3	6	0.55	0.56
15	25	2800	200	0.3	9	0.29	0.24
16	25	2800	400	0.6	4	1.05	1.07
17	25	2800	400	0.6	6	0.70	0.65
18	25	2800	400	0.6	9	0.42	0.42
19	40	1200	200	0.3	4	1.07	1.12
20	40	1200	200	0.3	6	0.75	0.77
21	40	1200	200	0.3	9	0.65	0.68
22	40	1200	400	0.3	4	1.40	1.37
23	40	1200	400	0.3	6	0.95	0.97
24	40	1200	400	0.3	9	0.75	0.80
25	40	2000	200	0.6	4	0.90	0.93
26	40	2000	200	0.6	6	0.65	0.60
27	40	2000	200	0.6	9	0.42	0.39
28	40	2000	400	0.6	4	1.35	1.39
29	40	2000	400	0.6	6	1.00	1.05
30	40	2000	400	0.6	9	0.52	0.56
31	40	2800	200	0.3	4	0.87	0.85
32	40	2800	200	0.3	6	0.45	0.43
33	40	2800	200	0.3	9	0.28	0.27
34	40	2800	400	0.6	4	0.99	0.92
35	40	2800	400	0.6	6	0.60	0.56
36	40	2800	400	0.6	9	0.36	0.39
37	60	1200	200	0.3	3	1.35	1.32
38	60	1200	200	0.3	6	0.95	0.92
39	60	1200	200	0.3	9	0.71	0.69
40	60	1200	400	0.3	3	1.52	1.47
41	60	1200	400	0.3	6	1.03	1.00
42	60	1200	400	0.3	9	0.80	0.79
43	60	2000	200	0.6	3	1.05	1.01
44	60	2000	200	0.6	6	0.70	0.74
45	60	2000	200	0.6	9	0.46	0.48
46	60	2000	400	0.6	3	1.40	1.38
47	60	2000	400	0.6	6	1.05	1.00
48	60	2000	400	0.6	9	1.00	0.97
49	60	2800	200	0.3	3	0.95	0.98
50	60	2800	200	0.3	6	0.50	0.45
